# GLUT1 expression, lymphocyte distribution and CD3^+^ T-cell metabolic subsets as predictive markers of response to immunotherapy in advanced melanoma

**DOI:** 10.1186/s13046-025-03637-8

**Published:** 2026-01-20

**Authors:** Elizabeth C. Paver, Tuba N. Gide, Zarwa Yaseen, Paola Cornejo-Paramo, Peter Ferguson, Nigel G. Maher, Alexander M. Menzies, Matteo S. Carlino, Ines Pires da Silva, Jeff Holst, Georgina V. Long, Richard A. Scolyer, James S. Wilmott

**Affiliations:** 1 Melanoma Institute Australia, The University of Sydney, Sydney, Australia; 2 Department of Anatomical Pathology, ACT Pathology, Canberra Health Services, Canberra, Australia; 3 School of Medicine and Psychology, Australian National University, Canberra, Australia; 4 Charles Perkins Centre, The University of Sydney, Sydney, Australia; 5 Faculty of Medicine and Health, The University of Sydney, Sydney, Australia; 6 Royal North Shore Hospital, Sydney, Australia; 7 Mater Hospital, North Sydney, Australia; 8 Westmead and Blacktown Hospitals, Sydney, Australia; 9 Champalimaud Foundation, Lisbon, Portugal; 10 School of Biomedical Sciences, UNSW Sydney, Kensington, Australia; 11 Tissue Pathology and Diagnostic Oncology, Royal Prince Alfred Hospital, Sydney, Australia; 12 NSW Health Pathology, Sydney, Australia

**Keywords:** GLUT1, GLUT3, Metabolic tumor microenvironment, Melanoma, Immunotherapy, Spatial distribution, Multiplex immunofluorescence, Tumor-infiltrating lymphocytes

## Abstract

**Background:**

Glycolysis, commonly used by malignant tumors for energy production, results in acidification of the tumor microenvironment (TME) through the secretion and accumulation of lactic acid. Acidosis is a potent inhibitor of immune cell function and may therefore affect T-cell infiltration and the efficacy of immunotherapy. This study aimed to characterize the metabolic tumor microenvironment and its association with lymphocyte distribution in patients with advanced melanoma treated with immune checkpoint blockade (ICB) therapies.

**Methods:**

Pre-treatment formalin-fixed, paraffin-embedded metastatic melanoma specimens from 45 patients treated with anti-PD-1 ± anti-CTLA-4 ICB were included in this study. Patients with progression-free survival (PFS) ≥ 6mo were categorized as responders (*n* = 23), while non-responders had a PFS < 6mo (*n* = 22). Two custom multiplex immunofluorescence panels were developed to evaluate the expression and distribution of markers of a hypoxic microenvironment (CA9 and HIF1α), glycolysis (GLUT1 and GLUT3) and vessels (CD31) in relation to melanocytes (SOX10) and T lymphocytes (CD3).

**Results:**

GLUT1 + melanoma regions contained significantly lower proportions of CD3^+^ T-cells than GLUT1- regions (*p* < 0.0001). Responders displayed significantly higher proportions of intratumoral T-cells expressing GLUT1 (*p* = 0.049) and GLUT3 (*p* = 0.043) compared to non-responders. CD3^+^ T-cells co-expressing hypoxia-associated markers were present in higher proportions significantly closer to GLUT1^+^ melanoma cells in responders compared to non-responders (*p* < 0.05). Patients with higher proportions of CD3^+^ T-cells and CD3^+^CA9^+^ T-cells within the 20 µm distance to GLUT1^+^ melanoma cells had significantly longer progression-free survival (*p* = 0.0133 and *p* = 0.0378, respectively).

**Conclusions:**

Together, these findings support the hypothesis that the presence of glycolysis in melanoma (as inferred by increased GLUT expression) may affect the ability of T-cells to infiltrate tumors and function effectively. The results also suggest that the overall proportion and spatial distribution of GLUT^+^ T-cells, including those displaying evidence of adaptation to a hypoxic/acidic TME, may be relevant for responses to ICB therapy.

**Supplementary Information:**

The online version contains supplementary material available at 10.1186/s13046-025-03637-8.

## Contributions to the literature

This study integrates metabolic marker assessment with spatial immune profiling to show that increased tumor glycolysis, inferred by GLUT1 and GLUT3 expression, is associated with reduced T-cell infiltration. The proximity and proportion of T-cells relative to glycolytic tumor regions are associated with clinical outcome, with closer T-cell localization to GLUT-expressing melanoma cells correlating with improved progression-free survival. We further identify an association between response to immune checkpoint blockade and the metabolic phenotype of tumor-infiltrating lymphocytes, with higher proportions of GLUT1^+^ and GLUT3^+^ T-cells observed in responders. Together, these findings extend prior biomarker studies by providing tissue-level spatial context for metabolic-immune interactions in melanoma, and support further investigation of tumor and immune cell metabolism as potential contributors to immunotherapy response.

## Introduction

Immune checkpoint blockade (ICB) of the cytotoxic T-lymphocyte antigen 4 (CTLA-4) and programmed cell death protein 1 (PD-1) have significantly improved outcomes in advanced melanoma; however, they fail to elicit a durable response in a significant proportion of patients. It is therefore critical that mechanisms of resistance are identified so that alternative treatment strategies can be developed. The use of glycolysis for energy production is a common feature of malignant tumors, including melanoma, resulting in acidification of the tumor microenvironment (TME) through the secretion and accumulation of lactic acid [1–3]. Acidosis is a potent inhibitor of immune cell migration and function, including T-cell effector function, leading to anergy and apoptosis [4, 5]. An acidic TME may therefore represent a significant barrier to the efficacy of immunotherapy.

Glycolytic metabolism can be initiated in response to tissue hypoxia, with hypoxia-inducible factor 1α (HIF1α) upregulating the expression of glucose transporter type 1 (GLUT1), a member of a family of proteins that facilitate increased glucose uptake for glycolysis [1]. However, GLUT1 expression does not always signify a hypoxic microenvironment. Many malignant tumors preferentially utilize glycolysis even when oxygen is available (a process known as aerobic glycolysis, or the Warburg effect) [1], as the by-products of glycolysis can be used for anabolic reactions that support the rapid proliferation and growth of the neoplastic cells [6]. Increased expression of GLUT1 has been reported in a variety of tumors, including melanoma in which expression is often zonal and/or heterogeneous [7], reflecting variability in metabolic adaptation within the TME [7]. Normally, GLUT1 is expressed by a limited number of cell types, including activated T lymphocytes, erythrocytes, keratinocytes, and endothelial cells of the central nervous system [8, 9]. More recently, increased expression of other glucose transporters (such as GLUT3) has also been identified in melanoma [10].

Tissue hypoxia and HIF1α can also upregulate other genes to help promote cell survival. Carbonic anhydrase IX (CA9) is a transmembrane enzyme that regulates cellular pH by reversibly catalyzing the hydration of carbon dioxide, neutralizing the intracellular pH (supporting ongoing tumor cell function), and further contributing to the acidification of the extracellular TME [2, 11–14]. We recently identified upregulation of genes associated with hypoxia, including CA9, among a population of patients with melanomas resistant to anti-PD-1 therapy [15]. CA9 expression has been identified as a poor prognostic biomarker in a number of tumors, including melanoma [16–18].

It is well established that the presence of tumor-infiltrating lymphocytes (TILs) is a positive prognostic feature in melanoma, with a multitude of studies demonstrating that higher TIL levels are associated with improved prognosis [19–23]. Different methods for the pathological assessment of TILs have been proposed, including scoring TILs based on distribution (peripheral/diffuse, or focal/multifocal/diffuse) and density (absent/non-brisk/brisk, or mild/moderate/marked) [20, 21, 24, 25]. Often, however, lymphocytes within tumors are only distributed within very close proximity to small intratumoral vessels within thin bands of fibrous stroma, and still appear to be relatively ‘excluded’ from the surrounding tumor, a TIL pattern we describe in this study as ‘perivascular’ exclusion (in contrast to ‘peritumoral’ exclusion, where lymphocytes appear confined to the peripheral edge of the tumor). There is likely a degree of inter-observer variability in the assessment of such cases, as perivascular lymphocytes have traditionally not been included in the scoring of TILs [25], and therefore the TIL score may vary depending on whether the association between lymphocytes and small intratumoral vessels has been accounted for or not. Such cases may also present a challenge for quantitative image analysis tools, which often require the definition of specific zones (e.g. ‘tumor’ vs ‘stroma’) for cell quantification, and may not be able to account for narrow bands of perivascular ‘stroma’ within a larger tumor bed.

A variety of functional and mechanical factors have been shown to be associated with T-cell exclusion, including low tumor antigenicity, altered chemokine and cytokine signalling, defective recruitment of antigen presenting cells, impaired antigen presentation and T-cell activation, vascular dysfunction, extracellular matrix barriers, and the presence of certain genomic abnormalities [26, 27]. The apparent restriction of lymphocytes to within close proximity of vessels raises the possibility that metabolic factors within nests of tumor cells (such as acidosis or hypoxia) may also present barriers to T-cell infiltration [28]. Given the importance of a close spatial relationship between lymphocytes and melanoma cells for responses to immunotherapy [29], this potential metabolic barrier may therefore also represent a mechanism of resistance to standard-of-care immunotherapies.

In this study, we developed a customized multiplex immunofluorescence (mIF) panel to investigate the metabolic microenvironment and distribution of T-cells in a cohort of immune checkpoint treated patients with advanced melanoma. Multiplex staining, which allows for the application of multiple antibodies to the same tissue section, represents a sophisticated method for evaluating the cellular composition of tumors, the co-expression of multiple markers by individual cells, and the spatial relationships between cells and structures. Our panel included GLUT1 as a marker of increased glycolytic metabolism, HIF1α and CA9 as markers of hypoxia, as well as SOX10 and CD3 to help identify melanoma cells and T lymphocytes, respectively. An additional panel, which included GLUT3 and endothelial marker CD31 in addition to GLUT1, SOX10 and CD3, was then performed on a subset of cases with sufficient tissue. We aimed to identify associations between markers of hypoxia and glycolysis, T-cell distribution, and resistance to anti-PD-1-based immunotherapies, hypothesizing that increased expression of GLUT1, CA9 and HIF1α, indicative of an acidic/hypoxic microenvironment, would be associated with reduced tumor infiltrating lymphocytes and resistance to ICB therapies.

## Methods

### Patients and specimens

Patients with unresectable stage III-IV metastatic melanoma who received anti-PD-1 ± anti-CTLA-4 immunotherapy were retrospectively identified from the Personalised Immunotherapy Program (NCT06536257). Patients without an adequate formalin-fixed, paraffin-embedded (FFPE) tissue biopsy, and with less than 100 melanoma cells within the biopsy, were excluded from the study. The study included 45 patients (*n* = 23 responders; *n* = 22 non-responders) with advanced melanoma. Patients with a progression-free survival (PFS) of greater than or equal to 6 months were classified as responders, while patients with a PFS of less than 6 months were categorized as non-responders. Samples were acquired with written informed consent from all patients and the Melanoma Biospecimen Tissue Bank. This study was conducted in accordance with the Declaration of Helsinki, and with ethical approval from the Sydney Local Health District Human Research Ethics Committee (Protocol No. X15-0454 and HREC/11/RPAH/444).

### Hematoxylin and Eosin (H&E) evaluation

The hematoxylin and eosin-stained sections corresponding to the sections used for multiplex immunofluorescence were reviewed by a pathologist (ECP). The following features were assessed: site, tumor morphology (epithelioid, spindled, rhabdoid, desmoplastic, mixed), the presence of peritheliomatous growth (defined as any region of viable melanoma cells forming a cuff around a central vessel, with necrosis or incipient necrosis away from the vessel), mitotic rate per mm^2^, TIL grade (0 = absent, 1 = mild, 2 = moderate, 3 = marked), and TIL pattern (0 = absent, 1 = peritumoral exclusion, 2 = perivascular exclusion, 3 = inflamed/infiltrative). TIL patterns 0, 1 and 2 were considered patterns of lymphocyte exclusion. The TIL grade and TIL pattern were assessed by two pathologists (ECP and PF). Inter-observer variability was assessed using the Cohen’s kappa (κ) coefficient, with moderate agreement for TIL grade (κ = 0.56) and substantial agreement for TIL pattern (κ = 0.77), as interpreted based on Landis and Koch criteria [30]. Samples with discordant scoring (*n* = 19) were assessed by a third pathologist (NGM) and a consensus was reached.

### Multiplex immunofluorescence staining

Four micrometre (µm) FFPE melanoma sections mounted on Superfrost Plus slides (Thermo-Scientific) were heated in the oven at 65 °C for 1 h, deparaffinized in xylene and rehydrated in graded ethanols. Antigen retrieval was performed in pH 9 Heat Induced Epitope Retrieval (HIER) buffer in the Decloaking Chamber (Biocare Medical) at 110 °C for 10 min. Slides were cooled on the benchtop in 1X Tris-buffered saline with Tween 20 (TBST) for 10 min before commencing staining using an Autostainer plus (DAKO). Tissue sections were blocked with 3% hydrogen peroxide in TBST for 5 min, and then incubated with the antibody for GLUT1 (Cell Signaling, 1:4000) for 30 min. The antibody was detected using the Opal Polymer HRP Ms + Rb (Onestep) (Quanterix) detection system, before visualization using Opal650 TSA (1:100) for another 5 min. Subsequently, antigen retrieval was conducted again to prepare the slides for the next antibody. Using this method, all samples were stained sequentially with CD3 (Cell Marque, 1:1500) visualized with Opal570 TSA (1:100), SOX10 (Biocare Medical, 1:200) visualized with Opal690 TSA (1:100), CA9 (Cell Marque, 1:50) visualized with Opal520 TSA (1:100) and HIF1α (Abcam, 1:200) visualized with Opal620 TSA (1:100). Slides were counterstained with DAPI (1:2000) for nuclei visualization, and cover slipped using the ProLong® Diamond Antifade Mountant (Invitrogen). Sections of a lymph node melanoma sample were stained with each primary antibody as positive controls. A lymph node section without primary antibody treatment was used as a negative control.

Samples were stained with a second multiplex immunofluorescence panel in the same manner on the ONCORE Pro X Automated Slide Stainer (Biocare Medical). This included the sequential staining of FFPE tissue sections with CD31 (Cell Signaling, 1:1600) visualized with Opal570 TSA, GLUT1 (Cell Signaling, 1:4000) visualized with Opal620 TSA, GLUT3 (Cell Signaling, 1:4000) visualized with Opal520 TSA, SOX10 (Biocare Medical, 1:200) visualized with Opal690 TSA, CD3 (Cell Marque, 1:1000) visualized with Opal780 TSA and the DAPI counterstain.

### Multispectral image analysis

Slides were imaged using the Vectra 3.0 and multispectral fluorescent images visualized in Phenochart v.1.0.8 (Quanterix). High-resolution images (20 ×) of the entire tumor and peritumor were spectrally unmixed in inForm v.2.4.1 (Quanterix) and stitched into a single multispectral image for each tissue specimen. Quantitative image analysis was performed in HALO v.2.2 (Indica Labs). Each sample was assessed to remove areas of necrosis, folded tissue or artefact staining. The Random Forest tissue classifier algorithm was trained to recognize tumor and peritumor based on the presence or absence of SOX10. A separate Random Forest tissue classifier was trained to distinguish between GLUT1-positive and GLUT1-negative regions within each sample. Positivity for each individual marker was determined by optimized thresholds based on the staining intensity. Tumor cells were considered positive for CA9 and GLUT1 if they showed at least partial membranous staining, with positivity reported as the percentage of total tumor cells. Staining was also manually assessed by a pathologist (ECP). A 20 µm distance threshold was utilized to assess proportions of immune cells within close proximity to melanoma cells based on our previous study [29]. Samples required a minimum of 50 immune cells (including immune cell subsets) and 100 melanoma cells for inclusion in spatial analysis. Three samples with dedifferentiated melanoma were excluded from analyses relying on accurate identification of melanoma cells. Five samples with GLUT1 expression on erythrocytes alone were excluded from any GLUT1-specific analyses.

Slides for mIF Panel 2 were imaged using the PhenoImager HT (Quanterix) and spectrally unmixed using the onboard automated workflow. Quantitative image analysis was performed in HALO v.4.1 (Indica Labs). As with the previous panel, the Random Forest tissue classifier was trained to recognize tumor and peritumor based on the presence or absence of SOX10. Positivity for each individual marker was determined by optimized thresholds based on the staining intensity. Tumor cells were considered positive for GLUT3 if they showed at least partial membranous staining, with positivity reported as the percentage of total tumor cells.

### Statistical analysis

Fisher’s exact test with Benjamini and Hochberg multiple test corrections was used to compare categorical variables (including male versus female, anti-PD-1 monotherapy versus combination anti-PD-1 + anti-CTLA-4, normal versus elevated lactate dehydrogenase (LDH), BRAF V600 mutation positive versus wildtype/non-BRAF mutation, prior BRAF/MEKi versus none). Unadjusted and adjusted p values, as well as the odds ratios and 95% confidence intervals are included in Supplementary Table 1. The Kruskal–Wallis test with Benjamini and Hochberg multiple test corrections was used to compare proportions of different cell types between subcutaneous, lymph node and other sites of metastases. The Mann–Whitney U test, with Benjamini and Hochberg multiple test corrections where appropriate, was used to compare proportions of hypoxic populations with response to immunotherapy. Unadjusted and adjusted p values, as well as the W statistic are included in Supplementary Table 2. The false discovery rate was set as 0.05. The Wilcoxon signed-rank test was used to compare proportions of T-cells between GLUT1^+^ and GLUT1^−^regions within the same tissue specimens. Survival curves were estimated using the Kaplan–Meier method and the log rank test was performed to determine associations between hypoxic populations and PFS. The optimal cutoffs for Kaplan–Meier analyses were determined using Cutoff Finder [31]. All statistical analyses were conducted using GraphPad PRISM (PRISM Version 10.4.1). A p-value of less than 0.05 was considered statistically significant.

## Results

### Patient characteristics

This study included 45 patients (*n* = 23 responders, *n* = 22 non-responders) treated with either anti-PD-1 monotherapy (*n* = 22) or combined anti-PD-1 with anti-CTLA-4 therapy (*n* = 23) (Table 1). The median age of the overall cohort was 59 years (range 36–86), with responders being significantly older than non-responders (median age = 70 vs 54, adj. *p* = 0.0011). There were no significant differences between responders and non-responders regarding sex, treatment type (monotherapy vs combination), presence of BRAF *V600* mutations, history of previous targeted therapy, baseline LDH (normal vs elevated) or M stage.

**Table 1:** Clinicopathologic characteristics of patients treated with anti-PD-1 ± anti-CTLA-4 immunotherapy

	**Responders (*n* = 23)**	**Non-Responders (*n* = 22)**	**Total (*n* = 45)**	***p* value**
**Sex (M:F)**	16:7	14:8	30:15	0.83
**Age, median (range)**	70 (47—86)	54 (36—66)	59 (36–86)	*0.0011
**M Stage at entry, n (%)**				
M0/M1a/M1b	9 (39)	6 (27)	15 (33)	0.65
M1c/M1d	14 (61)	16 (73)	30 (67)	
**Treatment cohort, n (%)**				
Anti-PD-1	14 (61)	8 (36)	22 (49)	0.33
Anti-PD-1/Anti-CTLA-4	9 (39)	14 (64)	23 (51)	
**BRAF V600 mutation, n (%)**	3 (13)	11 (50)	14 (31)	0.06
**Previous BRAF/MEK inhibitor therapy, n (%)**	2 (9)	5 (23)	7 (16)	0.33
**Cutaneous primary, n (%)**	22 (96)	18 (82)	40 (89)	0.33
**Baseline LDH, n (%)**				
Elevated	6 (26)	10 (45)	16 (36)	0.33
Normal	17 (74)	12 (55)	29 (64)	
**Site of biopsy, n (%)**				
Lymph node	5 (22)	11 (50)	16 (36)	N/A
Subcutaneous	15 (65)	8 (36)	23 (51)	
Other	3 (13)	3 (14)	6 (13)	
**Mitotic rate, median (range)**	12 (2–26)	12.5 (4–36)	12 (2–36)	0.33
**Peritheliomatous growth, n (%)**	4 (17)	9 (41)	13 (29)	0.33
**Metabolic zonation, n (%)**	12 (52)	11 (50)	23 (51)	> 0.99

*Abbreviations: M* male, *F* female, *Anti-PD-1* anti-programmed cell death-1, *anti-CTLA-4* anti-cytotoxic T-lymphocyte antigen-4, *LDH* lactate dehydrogenase, *%* percentage

Fisher’s exact test or Mann–Whitney test adjusted p-values following Benjamini and Hochberg multiple test corrections are reported where appropriate

^*^p < 0.05

### Pathological evaluation of H&E staining

**Fig. 1:**
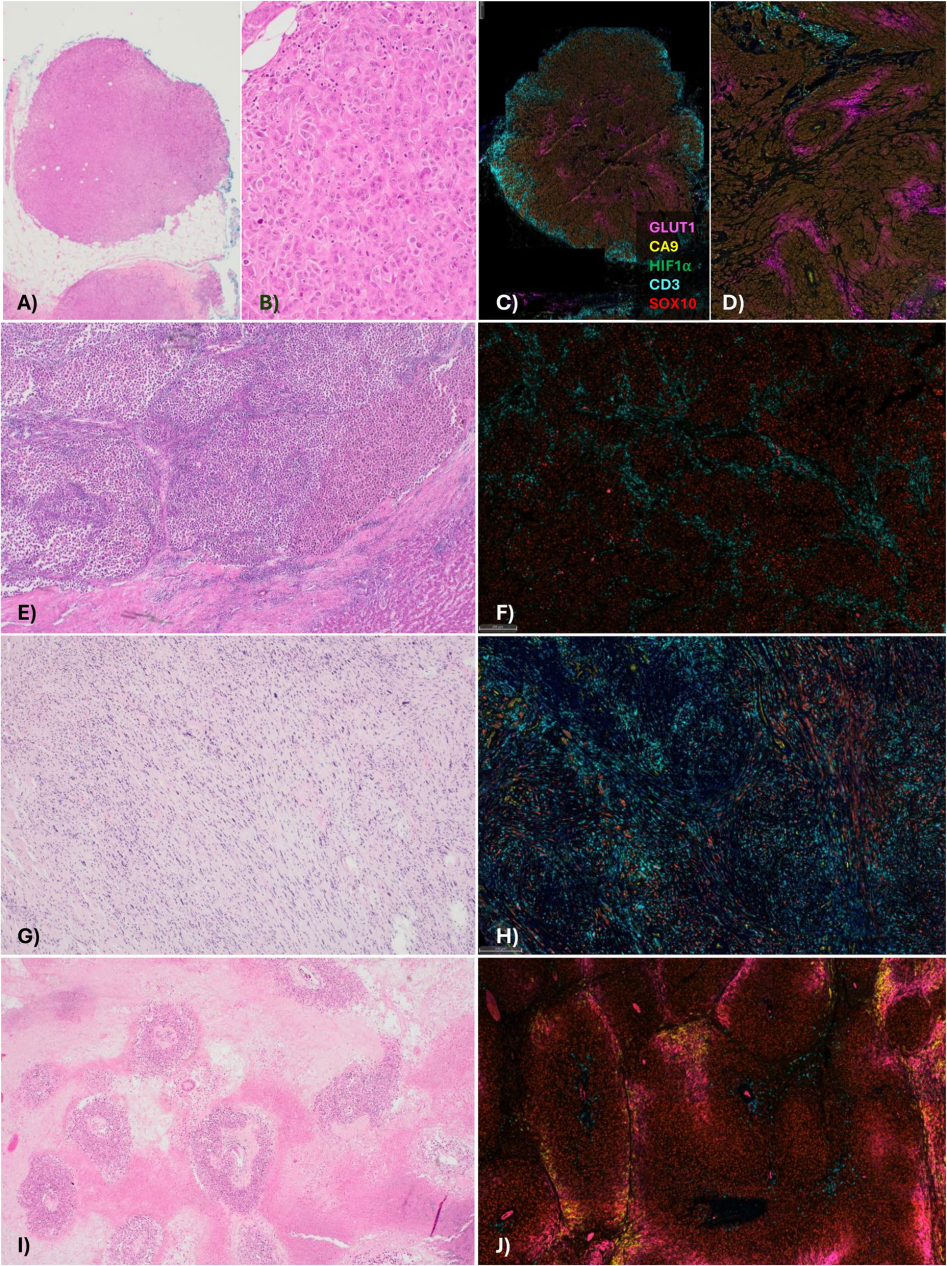
Examples of metastatic melanoma with different morphologies and patterns of lymphocyte distribution. **A**-**B** Metastatic melanoma showing solid growth with epithelioid and focally rhabdoid morphology, with peritumoral lymphocyte exclusion; **A**) H&E, 12.5 ×, **B**) H&E, 200 ×. **C**-**D** Corresponding multiplex immunofluorescence (IF), highlighting CD3^+^ cell exclusion at the periphery of the melanoma deposit, and showing focal central GLUT1 expression away from vessels, forming a pattern of ‘metabolic zonation’ not appreciated on H&E. Endothelial cells show CA9 positivity. **E**–**F** Liver metastasis, showing perivascular pattern of lymphocyte exclusion. **E** Lymphocytes preferentially locate along capillaries within the tumor, and do not infiltrate tumor islands (H&E, 40 ×). **F** Multiplex IF, highlighting presence of CD3^+^ lymphocytes in close proximity to vessels and absent from within tumor islands. There is no GLUT1 or CA9 expression, and only rare scattered HIF1α ^+^ cells. **G** Desmoplastic melanoma with atypical spindle cells and a diffuse/infiltrative pattern of TILs (H&E, 100 ×). **H** Multiplex IF of desmoplastic melanoma, highlighting diffuse infiltrate of CD3^+^ lymphocytes amongst scattered SOX10^+^ melanoma cells. The endothelial cells show CA9 positivity, and there are occasional scattered HIF1α cells. **I** Example of peritheliomatous growth pattern, with viable melanoma clinging to central vessels, and necrosis away from vessels (H&E, 20 ×). **J** Multiplex IF showing metabolic zonation, with CA9 and GLUT1 expression away from vessels, at interface with areas of incipient necrosis. CD3^+^ T-cells are only seen in close proximity to central vessels

We first examined the hematoxylin and eosin-stained sections to assess the morphological features of each tumor and identify possible confounders for downstream analysis. Our study included metastatic deposits in subcutaneous tissue (*n* = 23), lymph nodes (*n* = 16), lung (*n* = 3), submucosa (*n* = 1), liver (*n* = 1) and small intestine (*n* = 1). The melanomas demonstrated a spectrum of morphological appearances, with most cases showing epithelioid cytomorphology with variable spindle cell and/or rhabdoid areas (Fig. 1A-F). One case was predominantly desmoplastic (Fig. 1G-H). A peritheliomatous (pseudo-papillary) growth pattern was observed at least focally in 13 cases (29%) (Fig. 1I-J). The average cell size varied between tumors, ranging from 66.6 µm^2^ to 106.7 µm^2^. TILs were absent to mild in 32 cases (71%), moderate in 10 (22%) and marked in 3 cases (7%). Lymphocyte exclusion (TIL patterns 0–2) was observed in most cases, with lymphocytes only at the periphery of the tumor deposit (peritumoral exclusion, Fig. 1A-D) in 15 cases (33%), and present only in close proximity to vessels (perivascular exclusion, Fig. 1E-F, Supp Fig. 1 A) in a further 18 cases (40%).

### Expression and patterns of staining of CA9, GLUT1 and HIF1α in metastatic melanoma

**Fig. 2:**
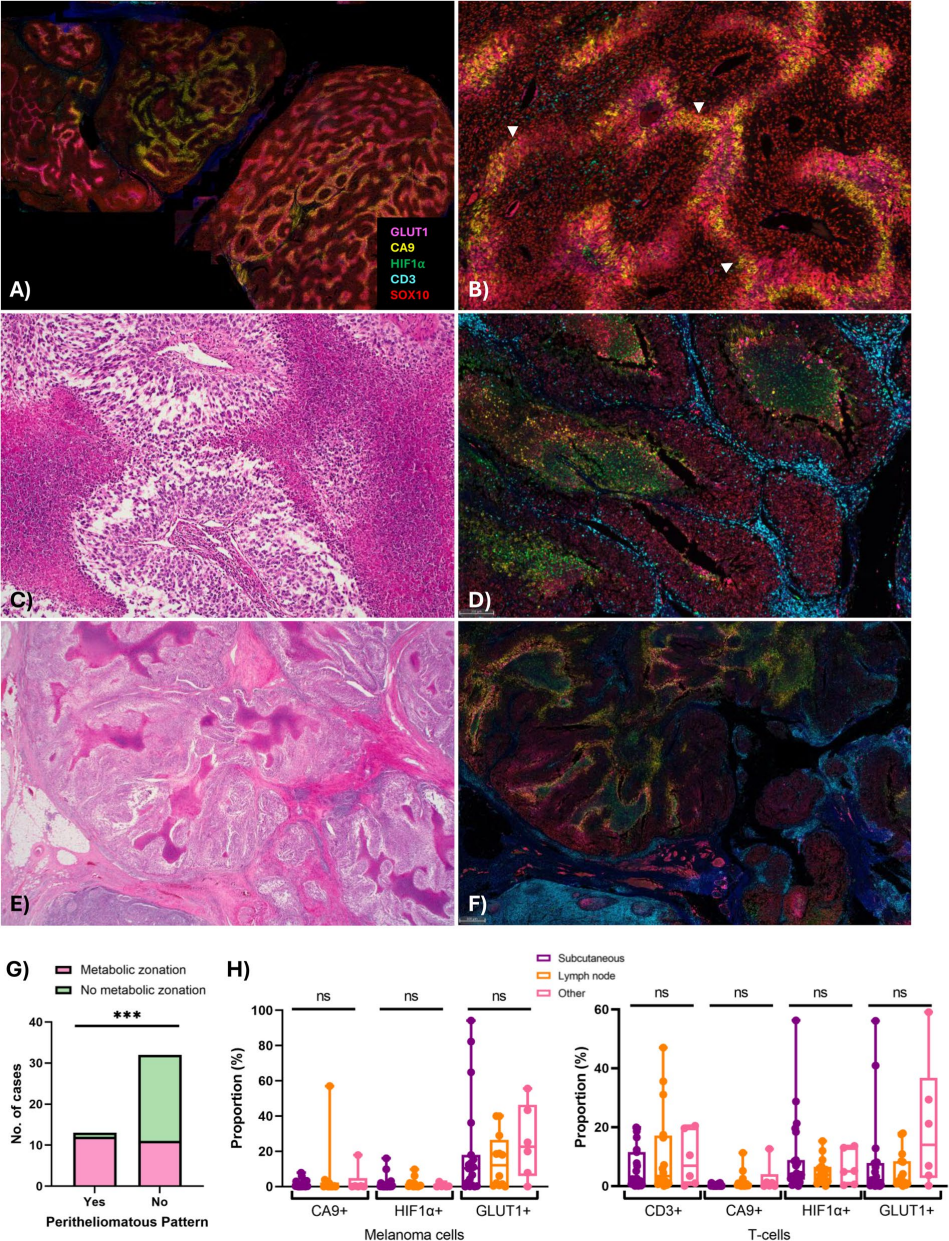
Expression of metabolic markers in metastatic melanoma specimens. **A**-**B** Lymph node replaced by metastatic melanoma;** A** Multiplex IF demonstrating a striking pattern of metabolic zonation, indicated by the ring-like pattern of CA9 (yellow) and GLUT1 (magenta). **B** Multiplex IF highlighting metabolic zonation (white arrows), with increased expression of CA9 and GLUT1 at the edge of the melanoma furthest from the vessels. **C** Peritheliomatous growth pattern, with viable melanoma clinging to central vessel, and peripheral necrosis (H&E, 100 ×).** D** HIF1α expression within areas of necrosis, with increased CA9 at tumor interface. CD3^+^ T-cells are excluded from tumor (SOX10^+^).** E** Melanoma with areas of geographic necrosis and focal pseudopapillary/peritheliomatous growth (H&E, 12.5 ×).** F** Multiplex IF demonstrating prominent zonation, with increased CA9 expression on tumor cells at the interface with areas of necrosis, which contain increased HIF1α. **G** Bar graph revealing significant association between a peritheliomatous growth pattern and metabolic zonation. **H** Box plots showing no significant differences in expression of metabolic markers on melanoma or T-cells between sites of disease. Error bars represent median ± 95% CI. ***p < 0.001, ns – non-significant

We next investigated the protein expression of the metabolic markers via quantitative image analysis of the multiplex immunofluorescence staining. Focal membranous CA9 expression was observed on melanoma cells, with 32% of cases (13/41) showing > 1% expression (Fig. 2A-B). Membranous expression of GLUT1 was also identified on tumor cells (though expression was often both membranous and cytoplasmic), with 76% of cases (28/37) showing > 1% expression (Fig. 1C-D). GLUT1 expression was also observed on erythrocytes and a subset of lymphocytes. There were no significant associations between tumor expression of CA9 or GLUT1 (≥ 1%) and patient clinical characteristics including gender, age, M stage or mutation status (Supp Tables 3 and 4). A ‘zonal’ pattern of CA9, GLUT1 and HIF1α expression was present in 23/45 cases (51%), with a gradient of increasing expression with distance from vessels, strongest at the interface between viable tumor and necrosis/incipient necrosis (Fig. 2A-F). The zonal pattern of CA9 expression was significantly associated with a peritheliomatous pattern of growth (*p* = 0.0006) (Fig. 2B-D, 2G). Expression of CA9 was seen in the endothelium of vessels within tumor in 15 cases (34%), though was often patchy and of variable intensity (Fig. 1D). Expression of HIF1α was observed on scattered immune cells including neutrophils, histiocytoid cells and lymphoid cells, most concentrated in areas of necrosis (Fig. 2C-F, Supp Fig. 1B). There were no significant differences in CA9, HIF1α and GLUT1 expression on melanoma cells or T-cells between different sites of metastasis (Fig. 2H).

### Expression of CA9 and GLUT1 in normal structures

The expression of CA9 and GLUT1 was also examined in normal tissue adjacent to the tumor regions. Expression of CA9 was observed in superficial squamous epithelium (Supp Fig. 1 C), endothelial cells of small vessels within lymph nodes (Supp Fig. 1D), as well as in bile duct epithelium and small intestinal crypts. GLUT1 was strongly positive in erythrocytes and on perineurium, and also showed weak zonal expression within germinal centres of lymph nodes (Supp Fig. 1D).

### GLUT1 expression on T-cells is associated with response to anti-PD-1-based therapies

**Fig. 3:**
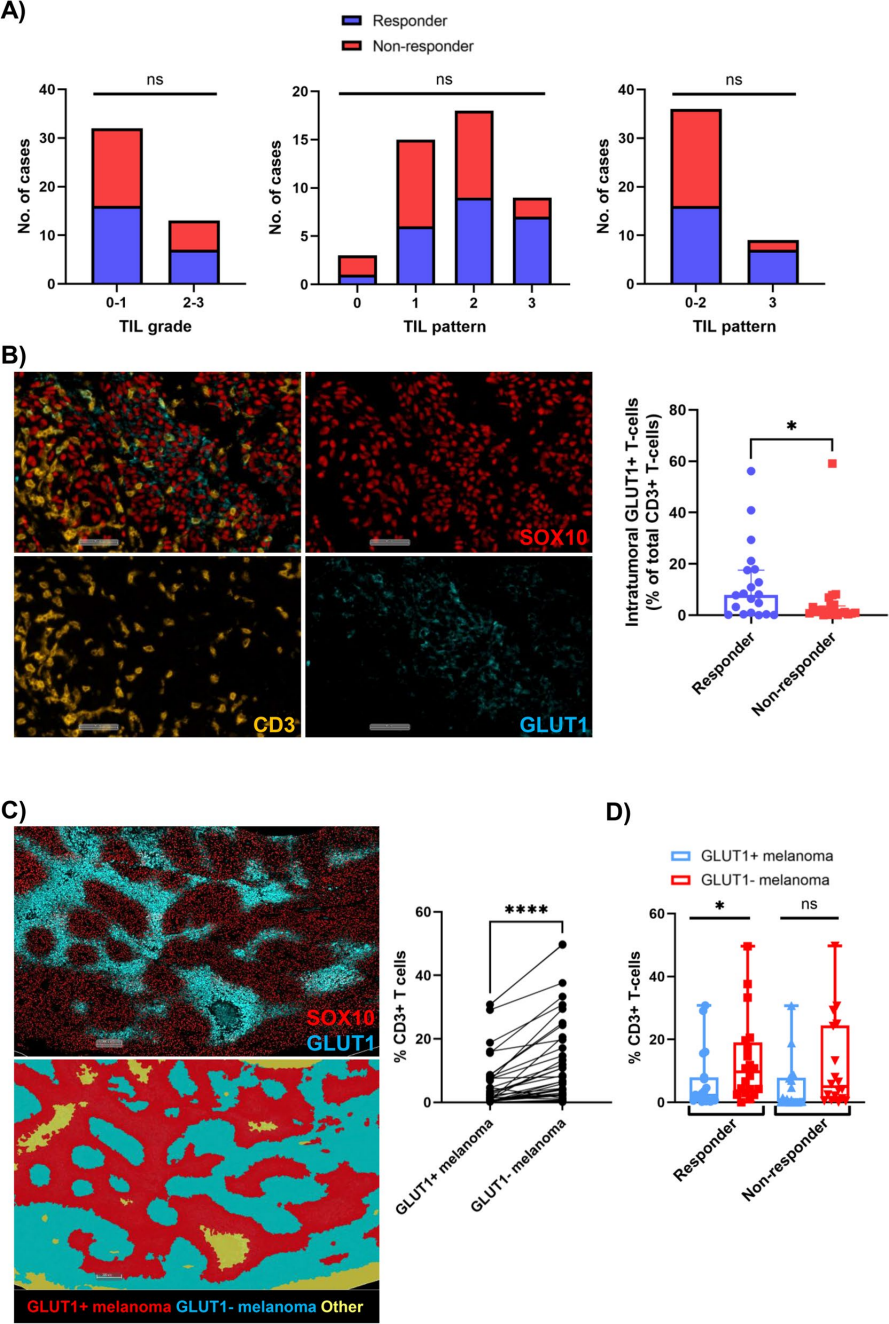
Associations between CD3^+^ T-cells, GLUT1 expression and immunotherapy response. **A** Bar plots illustrating no association between TIL grade (0 = absent, 1 = mild, 2 = moderate, 3 = marked) or TIL pattern (0 = absent, 1 = peritumoral exclusion, 2 = perivascular exclusion, 3 = inflamed/infiltrative) and response. **B** Representative images showing GLUT1^+^ staining on melanoma and T-cells, and box plots showing significantly higher proportion of GLUT1^+^ T-cells in responders compared to non-responders.** C** Comparison of the proportion of CD3^+^ T-cells in GLUT1^+^ tumor regions compared to GLUT1^−^ regions within the same samples. GLUT1^−^ melanoma has significantly higher proportions of CD3^+^ T-cells compared to GLUT1^+^ melanoma regions. **D** Comparison of the proportion of CD3^+^ T-cells in GLUT1^+^ regions compared to GLUT1^−^ regions in responders and non-responders. Responders have significantly higher proportions of CD3^+^ T-cells in GLUT1^−^ regions compared to GLUT1^+^ regions. Error bars represent median ± 95% CI. *p < 0.05, ****p < 0.0001, ns – non-significant

There were no significant differences in TIL grade, individual TIL patterns (Fig. 3A) or overall proportion of CD3^+^ T-cells between responders and non-responders (*p* > 0.05). While a higher proportion of non-responders exhibited a lack of lymphocyte infiltration (cases showing TIL patterns 0–2) compared to responders (91% vs 70%, respectively), this did not reach significance in this cohort (*p* = 0.1346; Fig. 3A). There was also no association between mitotic rate, peritheliomatous growth or the presence of metabolic zonation with response (*p* > 0.05; Table 1).

Responders had a significantly higher proportion of intratumoral CD3^+^GLUT1^+^ T-cells compared to non-responders (*p* = 0.049; Fig. 3B). There were no significant associations between the proportions of CA9^+^ or HIF1α^+^ T-cells in either the peritumoral or intratumoral regions (*p* > 0.05; Supp Fig. 2 A). Furthermore, there were no significant differences in melanoma expression of CA9, HIF1α or GLUT1 between responders and non-responders (*p* > 0.05; Supp Fig. 2 A and Supp Table 5).

### CD3^+^ T-cells are excluded from GLUT1^+^ melanoma regions

Evaluation of the staining pattern of the immune and glycolytic markers showed that CD3^+^ lymphocytes appeared to be excluded from areas of GLUT1 expression (Fig. 1C-D). Therefore, to further investigate the relationship between CD3^+^ T-cells and GLUT1^+^ melanoma cells, we trained a tissue classifier within a HALO AI algorithm to detect GLUT1 + and GLUT1- melanoma (SOX10^+^) regions (Fig. 3C). Analysis of the proportions of CD3^+^ T-cells within these regions revealed that there were significantly higher proportions of CD3^+^ T-cells in GLUT1-negative melanoma regions compared to GLUT1-positive regions within the same tumor, both overall (*p* < 0.0001; Fig. 3C) and in the responder group (*p* = 0.0334; Fig. 3D), with a similar (but non-significant) trend identified in non-responding patients (*p* = 0.0509; Fig. 3D).

### Spatial proximity of CD3^+^ T-cell subsets to GLUT1^+^ melanoma cells is significantly associated with response and progression-free survival

**Fig. 4:**
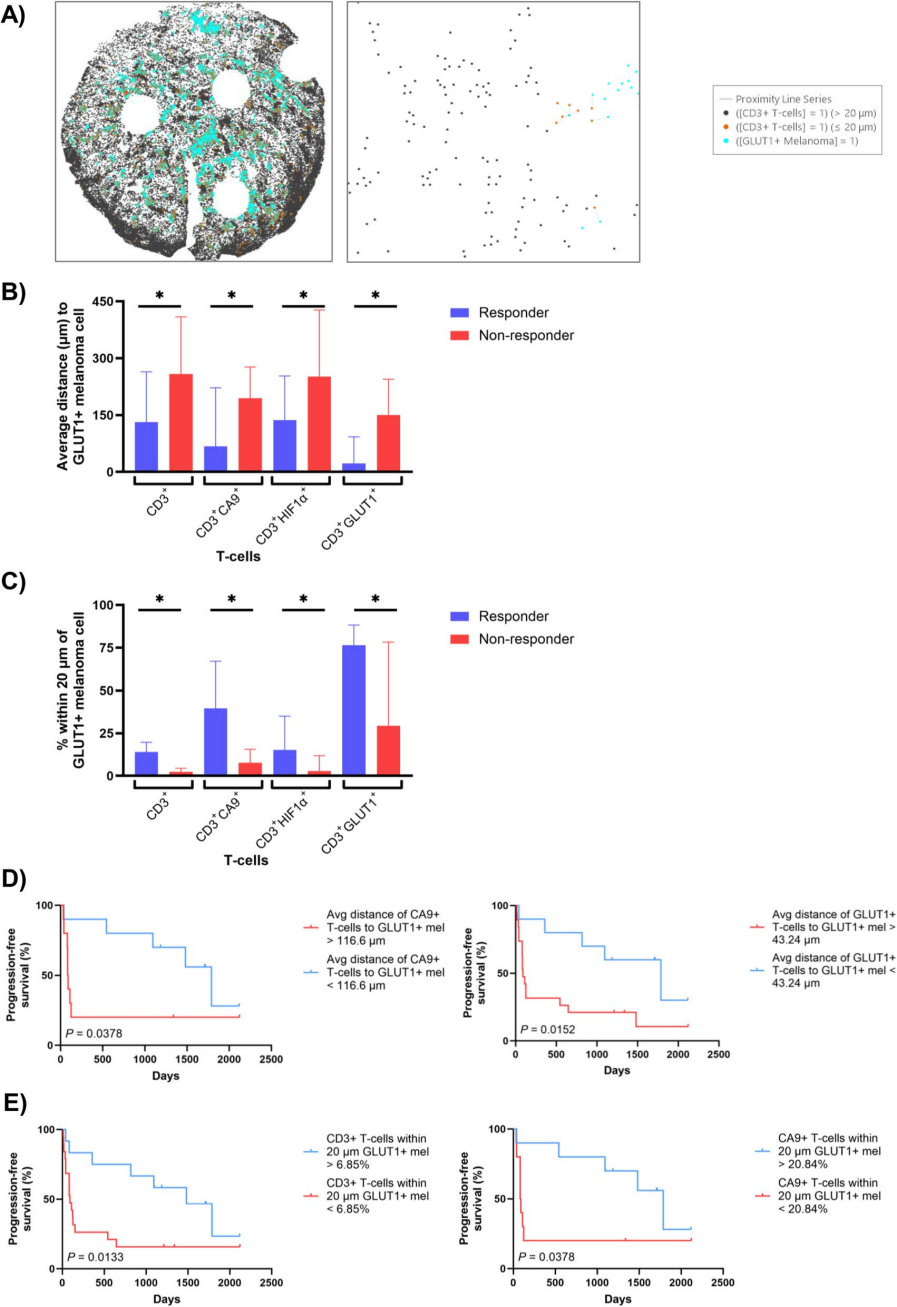
Associations between spatial locations of CD3 + T-cell subsets and clinical outcomes. **A** Representative image of spatial plot from responding patients showing distances between CD3^+^ T-cells and GLUT1^+^ melanoma cells. Blue dots indicate GLUT1^+^ melanoma cells, orange dots depict CD3^+^ T-cells located less than or equal to 20 µm from a GLUT1^+^ melanoma cell, and black dots show CD3^+^ T-cells located further than 20 µm from a GLUT1^+^ melanoma cell. The grey proximity line connects GLUT1^+^ melanoma cells to CD3^+^ T-cells within a 20 µm distance. **B** Bar plots illustrating significantly larger average distances between CD3^+^ T-cell subsets and GLUT1^+^ melanoma in non-responders compared to responders. **C** Bar plots illustrating significantly higher proportions of CD3^+^ T-cell subsets within 20 µm of a GLUT1^+^ melanoma cell in responders compared to non-responders. **D** Kaplan–Meier curves demonstrating significantly longer progression-free survival in patients with a shorter average distance between CD3^+^CA9^+^ T-cells or CD3^+^GLUT1^+^ T-cells and GLUT1^+^ melanoma. **E** Kaplan–Meier curves demonstrating significantly longer progression-free survival in patients with proportions of CD3^+^ T-cells and CD3^+^CA9^+^ T-cells within 20 µm of a melanoma cell that are above the cutoff. Error bars represent median ± 95% CI. *p < 0.05

Given our previous research revealing the importance of the spatial relationship between immune and melanoma cells for response to immunotherapy [29], we next assessed the average distances between CD3^+^ T-cells (including T-cell subsets expressing CA9, HIF1α and GLUT1) and melanoma cells, comparing response groups (Fig. 4A). We found that the CD3^+^ T-cells in responders were significantly closer to GLUT1^+^ melanoma cells compared to non-responders (adj. *p* = 0.0267; Fig. 4B). Responders also displayed significantly higher proportions of CD3^+^ T-cells within 20 µm of a GLUT1^+^ melanoma cell (adj. *p* = 0.0128; Fig. 4C). Regarding T-cell subsets, CD3^+^ T-cells that co-expressed CA9, HIF1α and GLUT1 were, on average, significantly closer to GLUT1^+^ melanoma cells in responding patients (adj. p = 0.0267, *p* = 0.0204, *p* = 0.0292, respectively; Fig. 4B), with responders also having significantly higher proportions of these subsets within 20 µm of GLUT1^+^ melanoma cells compared to non-responders (adj. *p* = 0.0345, *p* = 0.0411, *p* = 0.0345, respectively; Fig. 4C). There were no significant differences between responders and non-responders regarding distance of the immune cell subsets to all SOX10^+^ melanoma cells, CA9^+^ melanoma, or HIF1α^+^ melanoma (adj. *p* > 0.05; Supp Fig. 2B).

We then investigated the association between the spatial distribution of CD3^+^ T-cell subsets relative to GLUT1^+^ melanoma cells and PFS. A shorter average distance between CD3^+^CA9^+^ T-cells and GLUT1^+^ melanoma cells (*p* = 0.0378), and CD3^+^GLUT1^+^ T-cells and GLUT1^+^ melanoma (*p* = 0.0152) were significantly correlated with longer PFS (Fig. 4D). Furthermore, higher proportions of CD3^+^ T-cells and CD3^+^CA9^+^ T-cells within the 20 µm distance to GLUT1^+^ melanoma cells were significantly associated with longer PFS (*p* = 0.0133, *p* = 0.0378, respectively; Fig. 4E). There were no significant associations between the average distances of CD3^+^ T-cells and CD3^+^HIF1α^+^ T-cells to GLUT1^+^ melanoma and PFS (Supp Fig. 2 C), or the proportions of CD3^+^HIF1α^+^ T-cells and CD3^+^GLUT1^+^ T-cells within 20 µm of GLUT1^+^ melanoma cells and PFS (*p* > 0.05; Supp Fig. 2D).

### GLUT3 expression on T-cells is associated with response to anti-PD-1-based immunotherapies

**Fig. 5:**
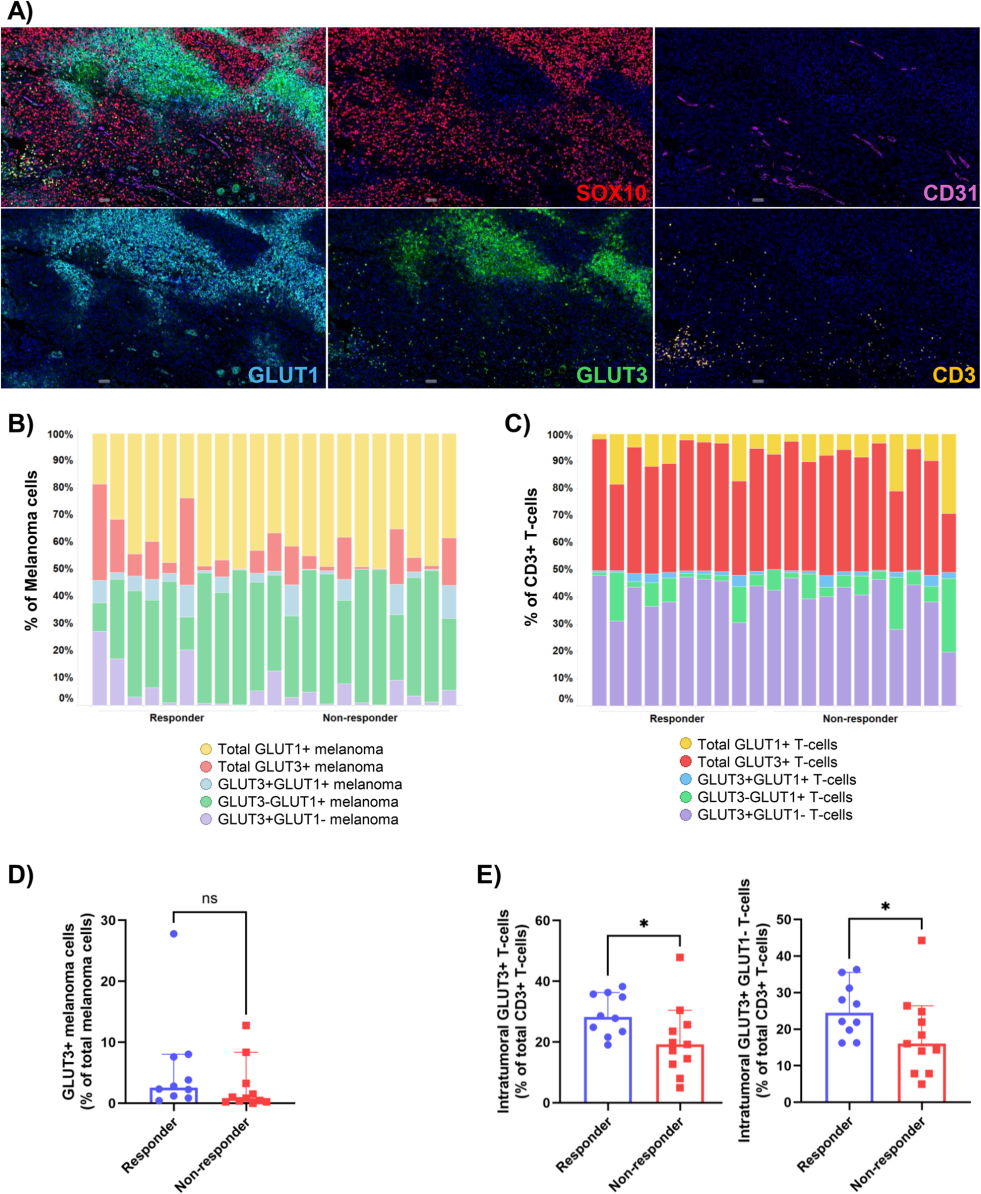
Associations between GLUT3 expression and immunotherapy response. **A** Representative multiplex image demonstrating expression of GLUT1 on SOX10^+^ tumor cells and CD31^+^ vessels, and GLUT3 expression on SOX10^+^ tumor cells and CD3^+^ T-cells.** B** 100% stacked bar plots illustrating GLUT1^+^ and GLUT3^+^ melanoma subsets as proportions of total SOX10^+^ melanoma cells. **C** 100% stacked bar plots illustrating GLUT1^+^ and GLUT3^+^ T-cell subsets as proportions of total CD3^+^ T-cells. **D** Bar plot showing no significant difference in the proportion of GLUT3^+^ melanoma between responders and non-responders.** E** Bar plots demonstrating significantly higher proportions of intratumoral GLUT3^+^ T-cells and GLUT3^+^GLUT1^−^ T-cells in responders compared to non-responders. Error bars represent median ± 95% CI. *p < 0.05, ns – non-significant

To further investigate the relationship between tumor glycolysis, T-cells and vascular distribution, we performed additional multiplex immunofluorescence staining on a subset of 21 patients with sufficient tissue (*n* = 10 responders, *n* = 11 non-responders) using a panel including GLUT1, GLUT3, CD31, CD3 and SOX10 (Fig. 5A and Supp Table 6). Membranous expression of GLUT3 was identified on > 1% of tumor cells in 62% of cases (13/21), showing a similar zonal pattern to GLUT1. Cytoplasmic GLUT3 expression was also observed on > 1% of CD3^+^ T-cells, in all cases (21/21). Co-expression of GLUT1 and GLUT3 on melanoma cells was observed in 43% of cases (9/21), and co-expression on T-cells in 71% of cases (15/21). Ninety percent of patients (19/21) had higher proportions of GLUT1^+^ melanoma compared to GLUT3^+^ melanoma (Fig. 5B), with melanoma cells showing a higher GLUT1 intensity compared to GLUT3 (Supp Fig. 3 A). In contrast, nearly all patients (20/21; 95%) had higher proportions of GLUT3^+^ T-cells compared to GLUT1^+^ T-cells (Fig. 5C). GLUT3 expression on melanoma was not associated with response (*p* = 0.099; Fig. 5D). However, responders had a significantly higher proportion of intratumoral CD3^+^GLUT3^+^ T-cells and CD3^+^GLUT3^+^GLUT1^−^ T-cells compared to non-responders (adj. *p* = 0.0430 and *p* = 0.0430, respectively; Fig. 5E). While there was a trend towards higher peritumoral CD3^+^GLUT3^+^ and GLUT3^+^GLUT1^−^ T-cells in responders compared to non-responders, this did not reach significance (adj. *p* = 0.0513 and *p* = 0.0513; Supp Fig. 3B). There were no significant associations between the proportions of intratumoral or peritumoral CD31^+^ endothelial cells and response (*p* > 0.05; Supp Fig. 3C).

### Spatial proximity of CD3^+^ T-cells to GLUT3^+^ melanoma cells is significantly associated with response

**Fig. 6:**
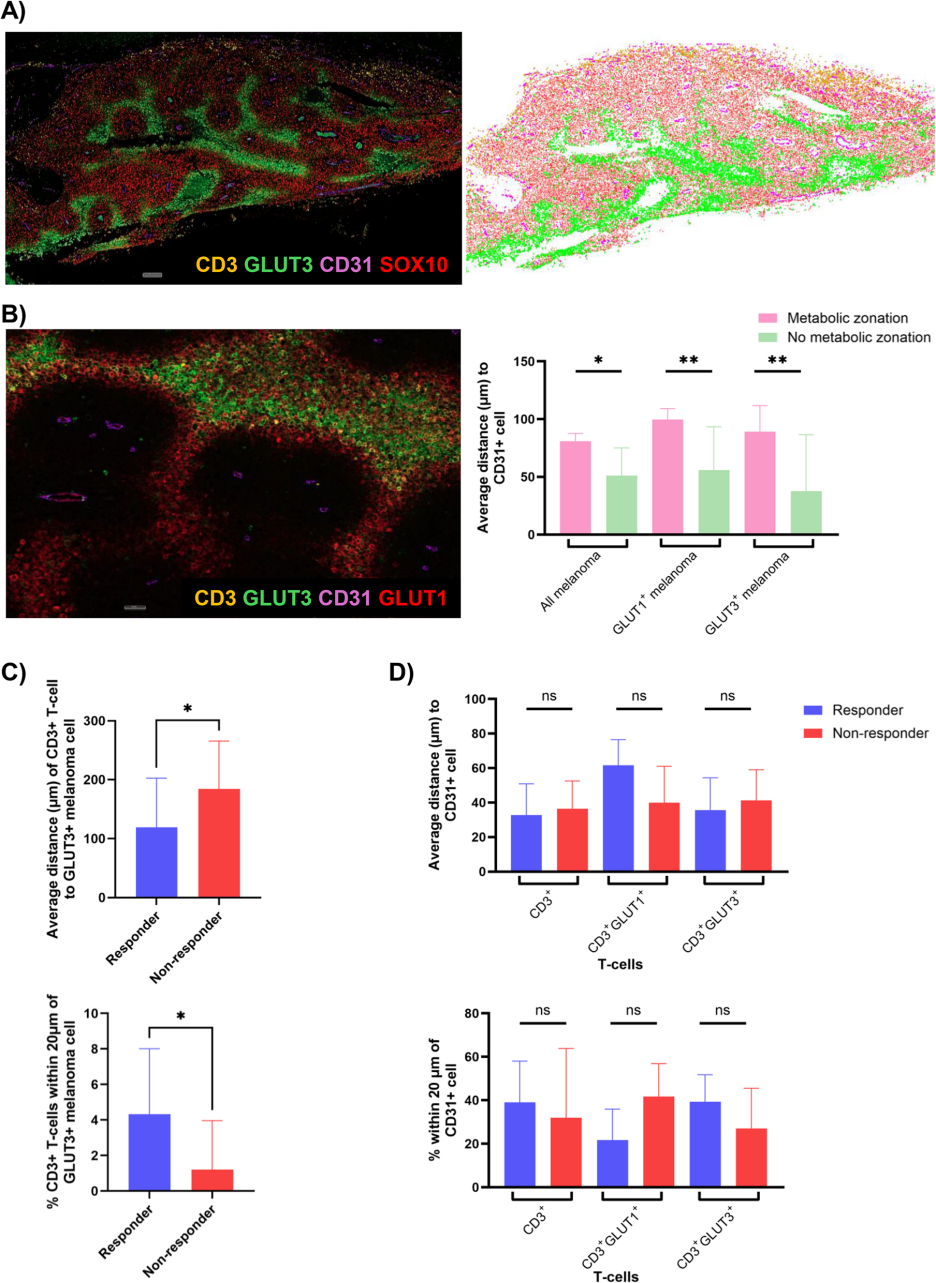
Associations between spatial locations of CD3^+^ T-cell subsets, CD31^+^ vessels and clinical outcomes. **A** Multiplex IF image and corresponding spatial plot showing locations of CD3^+^ T-cells (orange), GLUT3^+^ cells (green), CD31^+^ vessels (magenta) and SOX10^+^ melanoma cells (red).** B** Multiplex IF plot illustrating metabolic zonation pattern, with GLUT1^+^ and GLUT3^+^ melanoma located further away from CD31^+^ vessels. Bar plots showing all SOX10^+^ melanoma cells, GLUT1^+^ melanoma and GLUT3^+^ melanoma to be significantly further away from CD31^+^ vessels in tumors with metabolic zonation compared to those without metabolic zonation.** C** Bar plots showing CD3^+^ T-cells in closer proximity to GLUT3^+^ melanoma cells in responders compared to non-responders and significantly higher proportions of CD3^+^ T-cells within 20 µm of a GLUT3^+^ melanoma cell in responders.** D** Bar plots showing no significant differences in the average distances or spatial proximity of CD3^+^, CD3^+^GLUT1^+^ or CD3^+^GLUT3^+^ T-cells to CD31^+^ vessels between responders and non-responders. Error bars represent median ± 95% CI. *p < 0.05, **p < 0.01, ns – non-significant

Spatial analysis was subsequently performed to evaluate the distribution of T-cells, GLUT + melanoma and CD31^+^ vessels relative to each other (Fig. 6A-B). The average distance between CD31^+^ endothelial cells and melanoma cells (including all melanoma, GLUT1^+^ melanoma and GLUT3^+^ melanoma cells) was significantly greater in tumors with metabolic zonation (adj. *p* = 0.0125, *p* = 0.0015 and *p* = 0.0035, respectively; Fig. 6B). CD3^+^ T-cells in responders were significantly closer to GLUT3^+^ melanoma cells compared to non-responders (*p* = 0.0288; Fig. 6C). Responders also displayed significantly higher proportions of CD3^+^ T-cells within 20 µm of a GLUT3^+^ melanoma cell (*p* = 0.0185; Fig. 6C). However, there were no significant associations between the average distances of CD3^+^ T-cells to GLUT3^+^ melanoma and PFS (*p* = 0.4528; Supp Fig. 3D) or the proportions of CD3^+^ T-cells within 20 µm of GLUT3^+^ melanoma cells and PFS (*p* = 0.3949; Supp Fig. 3E). No significant differences were observed in the average distances of CD3^+^, CD3^+^GLUT1^+^ or CD3^+^GLUT3^+^ T-cells to CD31^+^ vessels between responders and non-responders (adj. *p* > 0.05; Fig. 6D). While there was a trend towards a lower proportion of CD3^+^GLUT1^+^ T-cells within 20 µm of a CD31^+^ vessel in responders compared to non-responders, this did not reach significance (adj. *p* = 0.0726; Fig. 6D).

## Discussion

Although ICB therapies have greatly improved outcomes for many patients with advanced melanoma, a significant proportion do not respond to treatment. A lack of lymphocyte infiltration is recognised as a major factor associated with ICB resistance, as again demonstrated in this study. We aimed to assess whether the metabolic tumor microenvironment, particularly the presence of tumour glycolysis and hypoxia, was related to T-cell infiltration, and to identify any relationship between these factors and response to ICB therapy.

Our results provide evidence that increased glycolysis in melanoma, as inferred by increased expression of GLUT1 and GLUT3, may impair T-cell infiltration, with GLUT+ zones containing significantly fewer CD3^+^ T-cells than GLUT- zones. Interestingly, though GLUT was often co-expressed with markers of hypoxia (CA9, HIF1α), no association was seen between lymphocyte distribution and expression of hypoxic markers, suggesting it is glycolysis itself that contributes to impaired T-cell infiltration, irrespective of whether due to hypoxia or the Warburg effect.

Indeed, there is accumulating evidence that glycolysis impacts both T-cell infiltration and function, and may also have implications for responses to immunotherapy. In fact, many of the previously identified factors associated with impaired T-cell infiltration and function either result in, or are a result of, increased glycolysis. For instance, the expression of glycolysis-related genes (such as *ALDOA)*has previously been shown to be negatively correlated with T-cell infiltration in melanoma, and promote resistance to T-cell-mediated anti-tumor activity [32]. Certain genomic aberrations, such as loss of *PTEN *and increased expression of beta-catenin, also result in upregulation of glycolysis, and are associated with impaired T-cell infiltration [33, 34], with melanomas lacking *PTEN *expression also showing reduced sensitivity to T-cell-mediated killing [32, 35]. Furthermore, elevated serum levels of lactate dehydrogenase (LDH, the product of enhanced glycolytic activity and tumor necrosis), seem to benefit less from checkpoint inhibitors than patients with normal LDH levels [36–38].

There are a number of mechanisms by which glycolysis may influence T-cell infiltration and function. Firstly, T-cell recruitment relies on, and is highly correlated with, the production of chemokines such as CXC ligands (CXCL) 9, 10 and 11 [39–41]. Melanomas with high glycolytic activity produce significantly lower concentrations of CXCL10, a critical T-cell-attracting chemokine produced largely by dendritic cells [32, 42]. Dendritic cells are also responsible for the priming and activation of specific effector T-cells through presentation of exogenous antigens on major histocompatibility complex class I (MHC-I) [43]. Tumor-derived lactic acid, a byproduct of glycolysis, significantly impairs dendritic cell differentiation and activation [42]. A strong correlation has previously been identified between CD8 gene transcripts and dendritic cell markers, suggesting that reduced T-cell activation and infiltration may be secondary to the defective recruitment and activation of dendritic cells [40]. Lactic acidosis has also been shown to impair capillary network formation and induce a mesenchymal/myofibroblast-like phenotype in endothelial cells, potentially impeding T-cell infiltration by contributing to vascular dysfunction and fibrosis [44].

Another possible mechanism is through the activity of the autotaxin (ATX)/lipophosphatidic acid (LPA) axis. ATX, encoded by *ENPP2,* is highly secreted by melanoma cells, with single-cell analysis of melanoma tumors identifying a negative correlation between intratumoral *ENPP2* expression and CD8^+^ T-cell infiltration [45]. ATX produces LPA, which has been shown to induce metabolic conversion towards aerobic glycolysis in cancer cells [46, 47]. LPA strongly suppresses chemotaxis and tumor infiltration of CD8^+^T-cells in melanoma-conditioned media, most likely through interaction with G protein coupled receptor LPAR6, expressed by melanoma TILs [45].

Furthermore, activated T-cells utilize glycolysis for proliferation and effector molecule production [48, 49]; therefore, reduced glucose availability (due to consumption by tumor cells) compromises the proliferative and functional capacity of T-cells [4, 5, 26]. The increased extracellular lactic acid (secreted by tumor cells) also alters the concentration gradient required by T-cells to secrete their own lactic acid byproduct, further impairing T-cell function [50, 51].

Despite the association with T-cell distribution, we did not find any association between GLUT expression on melanoma cells and response to treatment. It is important to note, however, that less than half our original cohort were able to undergo staining with GLUT3 (due to tissue constraints), and that our panels did not include other isoforms of GLUT which have more recently been identified in melanoma, such as GLUT4 and GLUT8 [52]. It is therefore possible that some tumors utilizing glycolysis were not identified, which may account for cases in our cohort that showed T-cell exclusion but were GLUT1 negative.

Whilst GLUT expression on melanoma cells did not differ between response groups, responders had significantly higher proportions of intratumoral GLUT1^+^ and GLUT3^+^ T-cells. T lymphocytes with high GLUT1 expression have been shown to preferentially acquire an effector phenotype, secreting significantly higher levels of interferon gamma (IFN-γ) than T-cells with low GLUT1 expression [8]. GLUT3 has higher glucose affinity and increased transport capacity than GLUT1, and is normally expressed in highly glucose-dependent tissues like the central nervous system [53]. GLUT3 is expressed by CD8^+^cytotoxic T-cells, where it has been shown in murine models to confer superior control of melanoma tumors and significantly improved survival [54]. However, a range of other T-cell subtypes also express GLUT1 or GLUT3, including naïve and exhausted T-cells, T helper 17 (Th17) cells [55], and tumor infiltrating regulatory T (Treg) cells [56]. The GLUT^+^ T-cell population in our study could not be further characterized, though the association with response may signify an effector function.

The significantly closer proximity of T-cells to GLUT1^+^ and GLUT3^+^melanoma in responders may reflect metabolic plasticity of the T-cell population in responders, possibly maintaining function in a low glucose/low oxygen environment by utilizing alternate nutrients/metabolites such as glutamate or fatty acid catabolism [52–54]. Interestingly, these lymphocytes also included high proportions co-expressing GLUT1, CA9 or HIF1α. The significance of the co-expression of hypoxic markers by T-cells is unclear, however recent single-cell and spatial transcriptomics data have also shown high expression of a spatially resolved GLUT1^+^/GLUT3^+^signature in hypoxic regions of breast cancer xenografts [57]. CA9 expression may represent an additional adaptive mechanism that facilitates lymphocyte survival in the acidic environment closer to GLUT^+^cells. Alternatively, it may indicate T-cell activation under hypoxic conditions, which results in significantly higher GLUT1 expression and enhanced T-cell effector function compared to T-cells activated under atmospheric oxygen conditions [8]. Both the shorter distance to GLUT^+^ melanoma cells, and increased density of CD3^+^ and CA9^+^ T-cells near GLUT1^+^ melanoma cells, were also associated with better PFS, suggesting that both the spatial distribution and metabolic phenotype of the lymphocyte population may be important for response.

There do not appear to be any consistent associations between GLUT1 expression and the molecular subtypes of melanoma, though data are relatively limited. Mutations affecting genes in the mitogen-activated protein kinases (MAPK) signalling pathway such as *BRAF* or *NRAS*, which are present in approximately two thirds of melanomas [58], ultimately affect the transcription of genes involved in glucose metabolism such as *GLUT1* through activation of signaling pathways such as PI3K/AKT/mTOR (which controls *PTEN*) and Wnt/βcatenin [59]. In colorectal carcinoma, there is some evidence that GLUT1 expression is increased in tumors harboring *KRAS* or *BRAF *mutations [60], supporting activation of the MAPK pathway as a mechanism for increased glycolysis. Interestingly, we found that a higher proportion of non-responders had *BRAF* V600 mutations compared to responders.

Limitations of our study include the relatively small number of patients for whom outcome data was available, and the heterogeneity of the population, which included a range of primary melanoma subtypes and treatments received (monotherapy vs combination therapy). Also, some rare melanoma subtypes, such as uveal melanoma (which typically responds poorly to ICB treatment), were not represented in our study, though it is interesting to note that a glycolysis-related gene signature is associated with worse prognosis in uveal melanoma [61], and furthermore that BAP1 inactivation (commonly found in uveal melanoma) promotes aerobic glycolysis [62]. The majority of the samples in our study were from cutaneous or nodal metastases, and it is uncertain whether the findings would be similar at other anatomical sites (such as liver or brain), which may have their own unique metabolic features. Furthermore, the multiplex platform only allowed a limited number of stains to be applied, and limited tissue was available for additional staining, therefore more comprehensive analysis of T-cell subtypes, GLUT isoforms, and other features of the tumor and TME was not possible. In addition, although our results implicate glycolysis as a mechanism contributing to T-cell exclusion, the observational design does not prove causation. Other factors, for example tumor antigenicity, upstream driver mutations, and architectural factors, may also affect T-cell infiltration, and further functional validation studies are required. Finally, three cases in our study needed to be excluded for tumor specific analyses as they contained dedifferentiated regions of melanoma that had lost SOX10 expression, and therefore could not be recognized as melanoma cells by the image analysis software. This is a potential pitfall of relying on AI-assisted technologies in tumor image analysis, and highlights the important role of the pathologist in cross-checking staining patterns and results with H&E sections.

## Conclusion

Overall, our results suggest that the metabolic microenvironment of melanoma, specifically the presence of glycolysis (as inferred by increased GLUT1/GLUT3 expression, with or without concurrent hypoxia), may play a critical role in the ability of immune cells to infiltrate tumors and function effectively. Furthermore, the overall proportion, spatial distribution and metabolic phenotype of the T-cell population appear to be important for response to immunotherapy.

## Supplementary Information

Supplementary Material 1.

Supplementary Material 2. Supplementary Figure 1.A) High power 40x multiplex immunofluorescence and pseudo-H&E image illustrating CD3+ T-cells in proximity to vessels, indicated by white arrows. B) High power 80x multiplex imageshowing GLUT1 expression on melanoma cells (white arrows), GLUT1 expression on CD3+ T-cells (blue arrows), and HIF1α expression on T-cells (green arrows). C) Representative multipleximage showing CA9 staining in squamous epithelium. D) Representative image illustrating GLUT1staining in a lymph node germinal center, and GLUT1 and CA9 staining of endothelial cells. Supplementary Figure 2. A) Bar plots showing no significant differences inCA9+/HIF1α+/GLUT1+ melanoma cells, or intratumoral CA9+/HIF1α+ T-cells betweenresponders and non-responders. B) Bar plots illustrating no significant differences in theaverage distances of CD3+ T-cell subsets to SOX10+ melanoma, CA9+ melanoma or HIF1α+melanoma between responders and non-responders. C) Kaplan–Meier curves demonstrating no significant improvement in progression-free survival in patients with a shorter average distance between CD3+ T-cells or CD3+HIF1α+ T-cells and GLUT1+ melanoma. D) Kaplan–Meier curves demonstrating no significant improvement in progression-free survival in patients with numbers of CD3+HIF1α+ T-cells or CD3+GLUT1+ T-cells within 20 μm of a melanoma cell that are above the cutoff. Error bars represent median ± 95% CI. ns – non-significant. Supplementary Figure 3. A) Scatter plot demonstrating higher expression of GLUT1 on SOX10+ melanoma cells compared to GLUT3. B) Bar plots showing no significant associations between the proportions of peritumoral GLUT3+ T-cells or GLUT3+GLUT1- T-cells and response. C) Bar plots showing no significant differences in CD31+ vessels between responders and non-responders. D) Kaplan–Meier curves demonstrating no significant improvement in progression-free survival in patients with a shorter average distance between CD3+ T-cells and GLUT3+ melanoma. E) Kaplan–Meier curves demonstrating no significant improvement in progression-free survival in patients with numbers of CD3+ T-cells within 20 μm of a GLUT3+ melanoma cell that are above the cutoff. Error bars represent median ± 95% CI. ns – non-significant.

## Data Availability

The data that support the findings of this study are available from the corresponding author, T.N.G., upon reasonable request.

## Abbreviations

ATX: Autotaxin
CA9: Carbonic anhydrase IX
CTLA-4: Cytotoxic T-lymphocyte antigen 4
FFPE: Formalin-fixed, paraffin-embedded
GLUT: Glucose transporter
H&E: Hematoxylin and Eosin
HIF1α: Hypoxia-inducible factor 1 subunit alpha
IFN-γ: Interferon gamma
LDH: Lactate dehydrogenase
LPA: Lipophosphatidic acid
ICB: Immune checkpoint blockade
MAPK: Mitogen-activated protein kinases
MHC-I: Major histocompatibility complex class I
mIF: Multiplex immunofluorescence
PD-1: Programmed cell death protein 1
PFS: Progression-free survival
Th17: T helper 17
TIL: Tumor-infiltrating lymphocytes
Treg: Regulatory T-cells
TME: Tumor microenvironment
