# Supplementary material for: GLUT1 expression, lymphocyte distribution and CD3+ T-cell metabolic subsets as predictive markers of response to immunotherapy in advanced melanoma
Source: J Exp Clin Cancer Res. 2026 Jan 20;45:51. doi: 10.1186/s13046-025-03637-8 (PMC12903248; doi:10.1186/s13046-025-03637-8)
Supplement: Supplementary file 1 — Supplementary Material 1. [file 13046_2025_3637_MOESM1_ESM.zip › Supplementary Table 3.docx]

| **SUPPLEMENTARY TABLE 3:** Key clinicopathological characteristics stratified by tumor GLUT1 expression | | | | |
| --- | --- | --- | --- | --- |
|  | **GLUT1 ≥ 1% (n = 28)** | **GLUT1 < 1%**  **(n = 9)** | **p value** |  |
| **Sex (M:F)** | 18:10 | 6:3 | >0.99 | |
| **Age, median (range)** | 61 (39 - 84) | 61 (54 - 76) | 0.97 | |
| **M Stage at entry, n (%)**  M0/M1a/M1b  M1c/M1d | 13 (46)  15 (54) | 1 (11)  8 (89) | 0.39 | |
| **Treatment cohort, n (%)**  Anti-PD-1  Anti-PD-1/Anti-CTLA-4 | 13 (46)  15 (54) | 6 (67)  3 (33) | 0.78 | |
| **BRAF V600 mutation, n (%)** | 6 (21) | 4 (44) | 0.50 | |
| **Previous BRAF/MEK inhibitor therapy, n (%)** | 2 (7) | 3 (33) | 0.39 | |
| **Cutaneous primary, n (%)** | 25 (89) | 8 (89) | >0.99 | |
| **Baseline LDH, n (%)**  Elevated  Normal | 10 (36)  18 (64) | 3 (33)  6 (67) | >0.99 | |
| **Site of biopsy, n (%)**  Lymph node  Subcutaneous  Other | 9 (32)  14 (50)  5 (18) | 3 (33)  5 (56)  1 (11) | N/A | |

Abbreviations: M – male; F – female; Anti-PD-1 – anti-programmed cell death-1; anti-CTLA-4 – anti-cytotoxic T-lymphocyte antigen-4; LDH – lactate dehydrogenase; % - percentage.

Fisher’s exact test or Mann-Whitney test adjusted p-values following Benjamini and Hochberg multiple test corrections are reported where appropriate.
