# Supplementary material for: GLUT1 expression, lymphocyte distribution and CD3+ T-cell metabolic subsets as predictive markers of response to immunotherapy in advanced melanoma
Source: J Exp Clin Cancer Res. 2026 Jan 20;45:51. doi: 10.1186/s13046-025-03637-8 (PMC12903248; doi:10.1186/s13046-025-03637-8)
Supplement: Supplementary file 1 — Supplementary Material 1. [file 13046_2025_3637_MOESM1_ESM.zip › Supplementary Table 4.docx]

| **SUPPLEMENTARY TABLE 4:** Key clinicopathological characteristics stratified by tumor CA9 expression | | | | |
| --- | --- | --- | --- | --- |
|  | **CA9 ≥ 1%**  **(n = 13)** | **CA9 < 1%**  **(n = 28)** | **p value** |  |
| **Sex (M:F)** | 9:4 | 19:9 | >0.99 | |
| **Age, median (range)** | 61 (37 - 86) | 60 (36 - 76) | >0.99 | |
| **M Stage at entry, n (%)**  M0/M1a/M1b  M1c/M1d | 5 (38)  8 (62) | 9 (32)  19 (68) | >0.99 | |
| **Treatment cohort, n (%)**  Anti-PD-1  Anti-PD-1/Anti-CTLA-4 | 6 (46)  7 (54) | 14 (50)  14 (50) | >0.99 | |
| **BRAF V600 mutation, n (%)** | 5 (38) | 7 (25) | >0.99 | |
| **Previous BRAF/MEK inhibitor therapy, n (%)** | 3 (23) | 3 (11) | >0.99 | |
| **Cutaneous primary, n (%)** | 12 (92) | 24 (86) | >0.99 | |
| **Baseline LDH, n (%)**  Elevated  Normal | 3 (23)  10 (77) | 12 (43)  16 (57) | >0.99 | |
| **Site of biopsy, n (%)**  Lymph node  Subcutaneous  Other | 5 (38)  7 (54)  1 (8) | 8 (29)  15 (54)  5 (18) | N/A | |

Abbreviations: M – male; F – female; Anti-PD-1 – anti-programmed cell death-1; anti-CTLA-4 – anti-cytotoxic T-lymphocyte antigen-4; LDH – lactate dehydrogenase; % - percentage.

Fisher’s exact test or Mann-Whitney test adjusted p-values following Benjamini and Hochberg multiple test corrections are reported where appropriate.
