# Supplementary figures and images for: GLUT1 expression, lymphocyte distribution and CD3+ T-cell metabolic subsets as predictive markers of response to immunotherapy in advanced melanoma
Source: J Exp Clin Cancer Res. 2026 Jan 20;45:51. doi: 10.1186/s13046-025-03637-8 (PMC12903248; doi:10.1186/s13046-025-03637-8)

A)

CD3 SOX10 DAPI

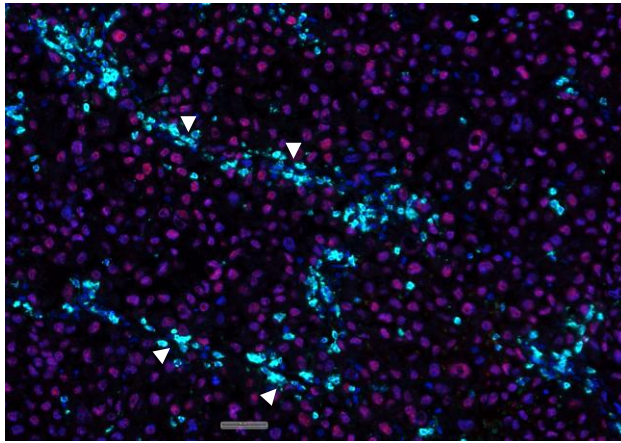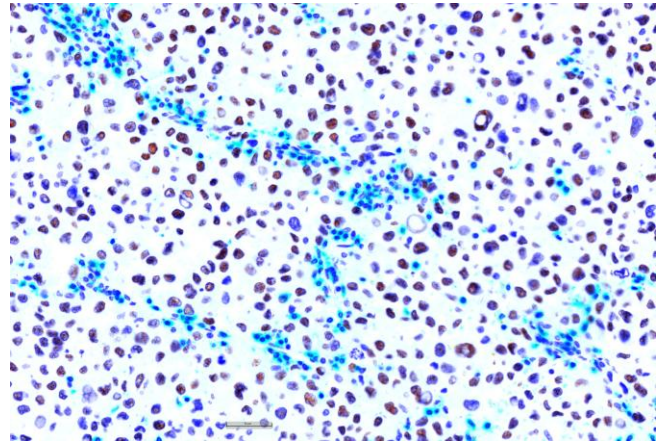

B)

CD3  
HIF1 $\alpha$   
GLUT1  
SOX10

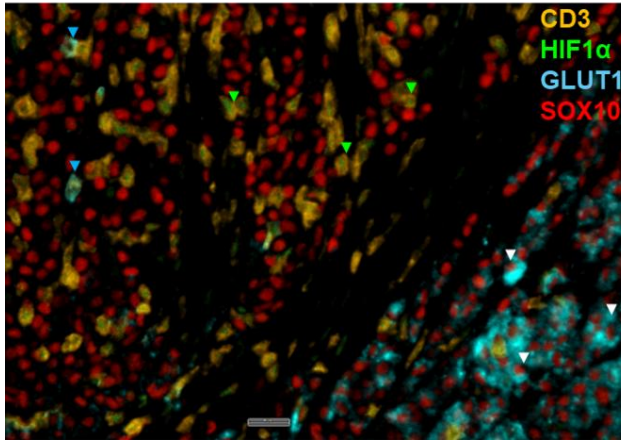

C)

CA9  
DAPI

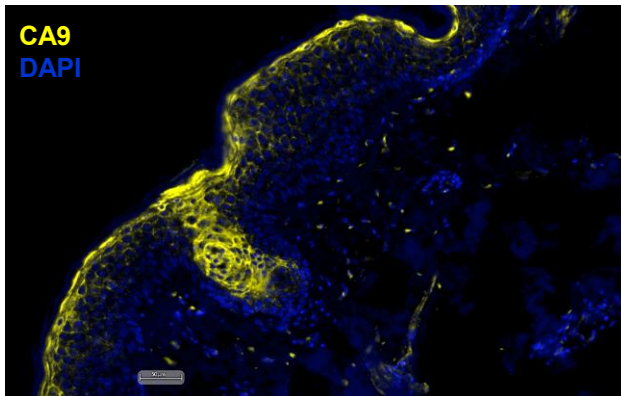

D)

CA9  
GLUT1  
DAPI

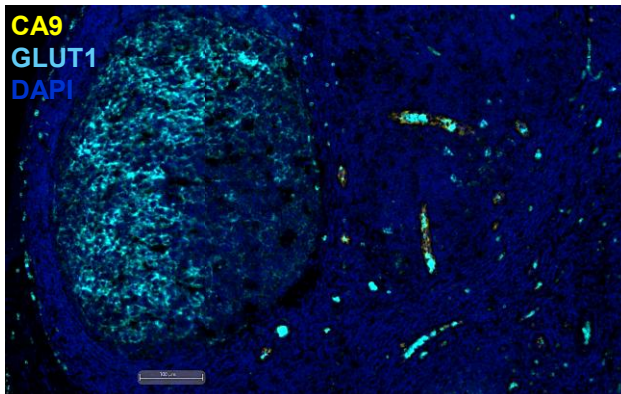

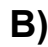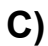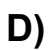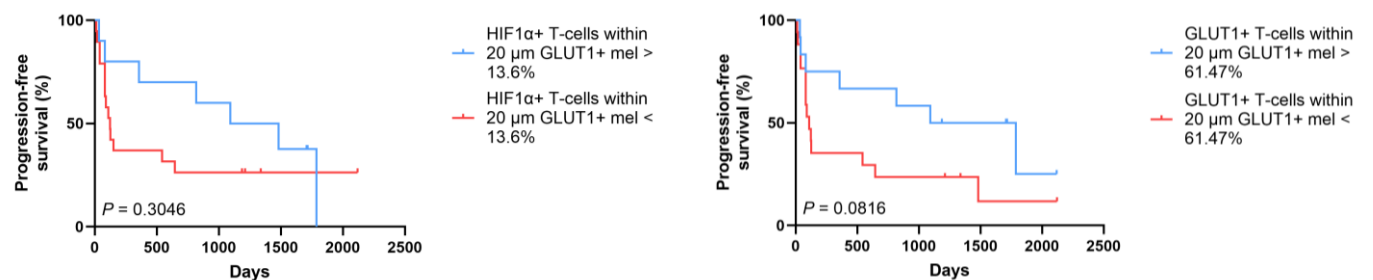

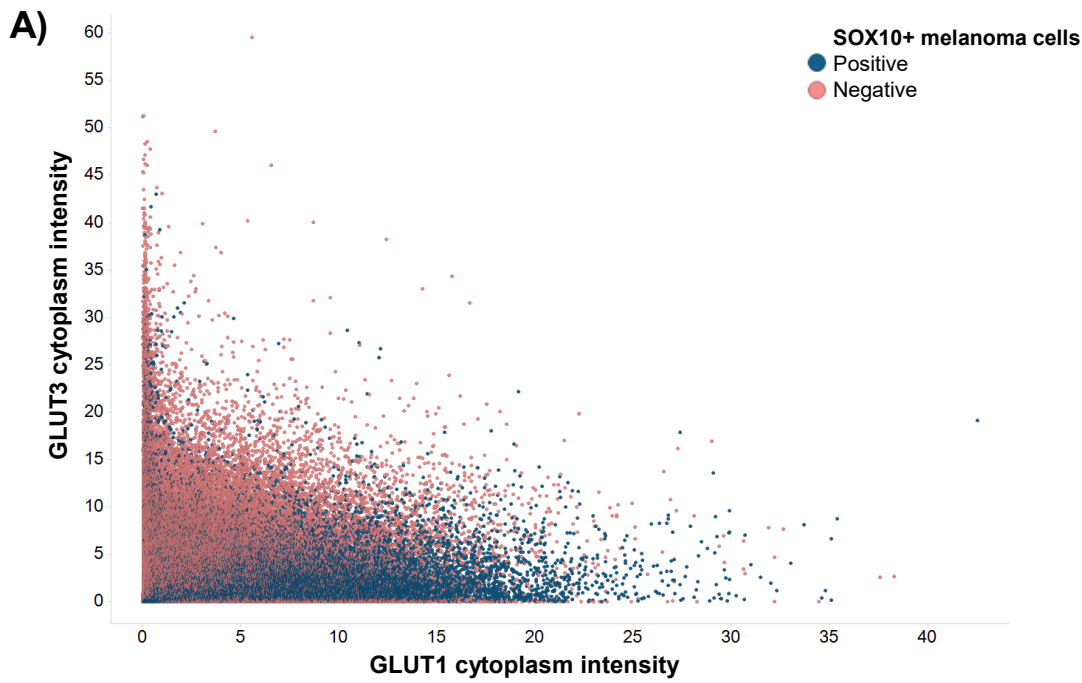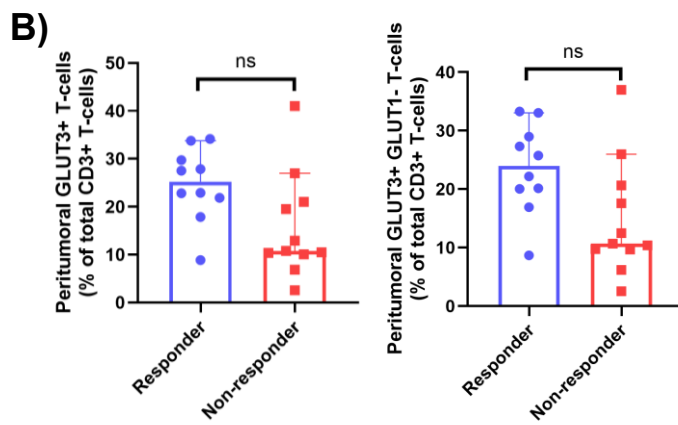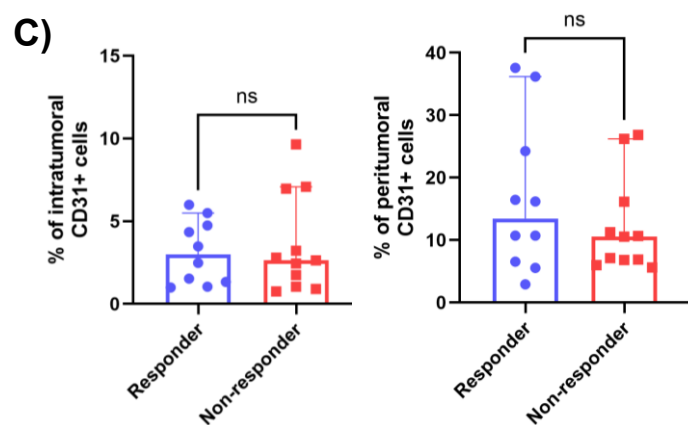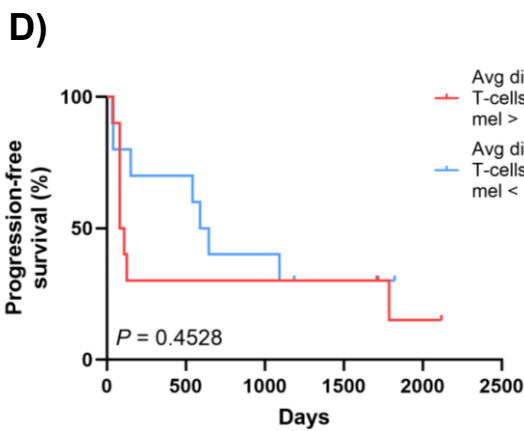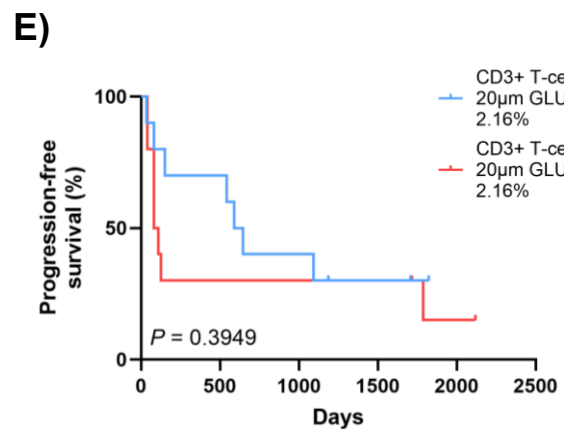

Supplement: Supplementary file 2 — Supplementary Material 2. Supplementary Figure 1.A) High power 40x multiplex immunofluorescence and pseudo-H&E image illustrating CD3+ T-cells in proximity to vessels, indicated by white arrows. B) High power 80x multiplex imageshowing GLUT1 expression on melanoma cells (white arrows), GLUT1 expression on CD3+ T-cells (blue arrows), and HIF1α expression on T-cells (green arrows). C) Representative multipleximage showing CA9 staining in squamous epithelium. D) Representative image illustrating GLUT1staining in a lymph node germinal center, and GLUT1 and CA9 staining of endothelial cells. Supplementary Figure 2. A) Bar plots showing no significant differences inCA9+/HIF1α+/GLUT1+ melanoma cells, or intratumoral CA9+/HIF1α+ T-cells betweenresponders and non-responders. B) Bar plots illustrating no significant differences in theaverage distances of CD3+ T-cell subsets to SOX10+ melanoma, CA9+ melanoma or HIF1α+melanoma between responders and non-responders. C) Kaplan–Meier curves demonstrating no significant improvement in progression-free survival in patients with a shorter average distance between CD3+ T-cells or CD3+HIF1α+ T-cells and GLUT1+ melanoma. D) Kaplan–Meier curves demonstrating no significant improvement in progression-free survival in patients with numbers of CD3+HIF1α+ T-cells or CD3+GLUT1+ T-cells within 20 μm of a melanoma cell that are above the cutoff. Error bars represent median ± 95% CI. ns – non-significant. Supplementary Figure 3. A) Scatter plot demonstrating higher expression of GLUT1 on SOX10+ melanoma cells compared to GLUT3. B) Bar plots showing no significant associations between the proportions of peritumoral GLUT3+ T-cells or GLUT3+GLUT1- T-cells and response. C) Bar plots showing no significant differences in CD31+ vessels between responders and non-responders. D) Kaplan–Meier curves demonstrating no significant improvement in progression-free survival in patients with a shorter average distance between CD3+ T-cells and GLUT3+ melanoma. E [file 13046_2025_3637_MOESM2_ESM.pdf]
